# Marine Organisms with Anti-Diabetes Properties

**DOI:** 10.3390/md14120220

**Published:** 2016-12-01

**Authors:** Chiara Lauritano, Adrianna Ianora

**Affiliations:** Department of Integrative Marine Ecology, Stazione Zoologica Anton Dohrn, 80121 Naples, Italy; adrianna.ianora@szn.it

**Keywords:** marine organisms, metabolic disorder, diabetes, microalgae, marine biotechnology, drug discovery

## Abstract

Diabetes is a chronic degenerative metabolic disease with high morbidity and mortality rates caused by its complications. In recent years, there has been a growing interest in looking for new bioactive compounds to treat this disease, including metabolites of marine origin. Several aquatic organisms have been screened to evaluate their possible anti-diabetes activities, such as bacteria, microalgae, macroalgae, seagrasses, sponges, corals, sea anemones, fish, salmon skin, a shark fusion protein as well as fish and shellfish wastes. Both in vitro and in vivo screenings have been used to test anti-hyperglycemic and anti-diabetic activities of marine organisms. This review summarizes recent discoveries in anti-diabetes properties of several marine organisms as well as marine wastes, existing patents and possible future research directions in this field.

## 1. Introduction

**Figure 1:**
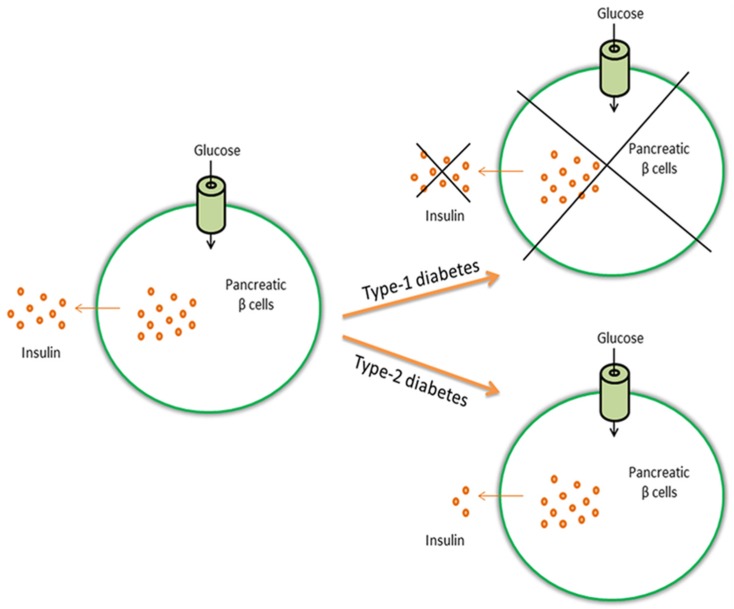
Glucose induces the release of insulin from pancreatic β cells. In type-1 diabetes, β cells are destroyed and insulin is not produced, whereas in type-2 diabetes the body does not produce enough insulin or cells do not react to insulin (insulin resistance).

Metabolic disorders (i.e., any of the diseases or disorders that disrupt normal metabolism) are common pathologies, and especially diabetes [1]. In 2013, it was estimated that over 382 million people throughout the world have diabetes and this number is expected to increase up to 500 million in 2030 [2] when it is expected that this disease will be the 7th leading cause of death [3]. Diabetes is usually caused by the interaction of genetic and environmental factors and is characterized by a lack of insulin secretion (relative and absolute) and insulin resistance, always leading to metabolism disorders of fat, protein and carbohydrate [4]. Insulin is a peptide hormone produced by beta cells of the pancreatic islets [2]. It has two essential functions without which the body would cease to function: (1) insulin stimulates glucose uptake and lipid synthesis; and (2) insulin inhibits the breakdown of lipids, proteins and glycogen, and also inhibits the glucose pathway (gluconeogenesis) [5,6,7]. Many people affected by diabetes will eventually have a series of diabetic complications like nephropathy, neuropathy, retinopathy, diabetic foot, ketoacidosis, and even increased risk of cardiovascular diseases and hypertension [4]. There are two types of diabetes, type-1 and type-2 (Figure 1), and also what is termed gestational diabetes that affects females during pregnancy. In type-1 diabetes, the beta cells are destroyed due to an autoimmune response and there is no insulin production [8]. What starts the autoimmune destruction is unknown, and may be due to a combination of genetic and environmental factors [9]. Type-1 diabetes is also referred to as insulin-dependent diabetes because patients need to take insulin injections for the rest of their life. In type-2 diabetes, the body does not produce enough insulin for proper functioning or the cells do not react to insulin (insulin resistance). In this case, patients can control the pathology by following a low calorie diet and exercising, even if they may need to take daily insulin injections or tablets. Type-2 diabetes is often associated to obesity and is related to eating high calorie diet and having a sedentary lifestyle. The occurrence of type-2 diabetes is more common, covering 90%–95% of all diabetes cases [10].

The complicated regulatory networks involved in the pathophysiology of diabetes are still not completely understood. There is evidence that inflammation processes and oxidative stress are at the basis and/or participate in the development of the disease [11]. For this reason, screening activities looking for anti-diabetic compounds include anti-inflammation and antioxidant tests, such as the inhibition of inflammatory mediators (e.g., tumor necrosis factor α (TNFα) or interleukin 6 (IL6)) and the activation of free radical detoxification enzymes/proteins (e.g., superoxide dismutase (SOD) and glutathione) [4,12,13]. In the next paragraph, we give an overview of common targets for anti-diabetes assays.

Because of the increasing number of diabetic patients and the limited number of anti-diabetic drugs, the search for new compounds, especially from marine sources, has attracted much interest from the scientific community. Marine bioresources have been shown to produce a number of novel scaffolds often with unusual skeletons [14,15]. Some commercially available marine compounds to treat other human pathologies [16,17] include anticancer drugs such as cytarabine (Cytosar-U^®^, Ara-C, DepoCyt^®^), isolated from the Caribbean sponge *Tethya crypta*, to treat acute myelocytic leukemia and non-Hodgkin’s lymphoma [16,18], trabectedin (Yondelis^®^), from the tunicate *Ecteinascidia turbinate*, approved for the treatment of tissue sarcomas and ovarian cancer [19], and Eribulin (Halaven^®^), from the sponge *Halichondria okadai* [20], for the treatment of metastatic breast cancer and advanced liposarcoma. There are also examples of marine compounds such as Ziconotide (Prialt^®^), isolated from the cone snail *Conus magus*, for the treatment of severe and chronic pain [21] and Vidarabine (Ara-A), from the sponge *Tethya crypta* [22] to treat herpes simplex infections. Interestingly, there is now also a terpene (Dysidine) extracted from the sponge *Dysidea villosa* that has entered preclinical trials for the treatment of diabetes [23]. Hence, there is great scope in the future to screen for anti-diabetic compounds from marine organisms.

In this review, we give an overview of the marine organisms that have shown anti-diabetes properties until now, provide a list of existing patents on these molecules and consider possible future research directions in this field.

## 2. Common Targets for Anti-Diabetes Assays

Anti-diabetes screenings include evaluation of the functioning of specific enzymes involved in sugar metabolism, in both rat models and patients (e.g., α-amylase, α-glucosidase, *N*-acetyl-glucosaminidase, aldose reductase, hexokinase, glucose-6-phosphatase, dipeptidyl peptidase IV, glucose transporter 4, and glycogen synthase kinase-3β) [24,25,26,27,28,29,30,31]. For instance, α-amylase and α-glucosidase are involved in the breakdown of ingested carbohydrates and their inhibition delays the absorption of glucose by acting as a possible strategy in the management of type-2 diabetes [32]. *N*-acetyl-glucosaminidase catalyzes the hydrolysis of glycosidic linkages as an exoglycosidase and releases *N*-acetyl-glucosamine from glycoprotein. This activity is markedly increased in patients with diabetes. Aldose reductase [33] is the first enzyme of the polyol pathway responsible for fructose formation from glucose, while hexokinase, glucose-6-phosphatase and dipeptidyl peptidase IV are involved in the metabolism of glucose. Sucrase and maltase are also involved in the metabolism of sugars, while glucose transporter 4 is involved in the transportation of glucose and is insulin-regulated [34]. Finally, glycogen synthase kinase-3β (GSK-3β) is a serine/threonine protein kinase that mediates the addition of phosphate molecules onto serine and threonine amino acid residues and has recently been implicated in a number of diseases, including type-2 diabetes, Alzheimer’s disease, inflammation and cancer [35].

Another common target for anti-diabetes testing is the protein tyrosine phosphatase 1B (PTP1B). This is an enzyme family that includes about 100 proteins that catalyze dephosphorylation of phosphotyrosine residues in protein substrates, such as the insulin receptor. PTP1B antagonizes insulin signaling by reducing the activation state of the insulin receptor kinase, thereby inhibiting post-receptor signaling in insulin responsive tissue. For this reason, this enzyme is associated with the development of type-2 diabetes [2,36,37,38]. Finally, clinical values are frequently analyzed in both diabetic rat models and/or patients, including blood glucose concentration, plasma insulin, blood pressure, triacylglycerol concentrations, total cholesterol, body weight and intestine histopathology [4,11,13,39,40,41,42]. These screenings mainly target possible compounds for the treatment of type-2 diabetes. Possible screenings for the more severe type-1 diabetes consist in identifying molecules able to protect pancreatic β cells (which produce insulin), by reducing inflammation and oxidative processes [2].

One of the most common effects associated with diabetes is retinopathy, a damage that occurs to the retina which causes rapid vision loss and can eventually lead to blindness [43]. Examples of targets to study diabetic retinopathy are the formation and accumulation of advanced glycation end-products (AGEs), expression levels of proteins/enzymes involved in abnormal neovascularization as well as the search for cell-based models. The formation of AGEs is a key pathophysiological process involved in diabetic retinopathy and blindness [43]. AGEs are generated from the glycation between sugars and proteins leading to molecule cross-linking and impairment. Several studies have used anti-glycoxidative activity as a test for anti-diabetes activity. The vascular endothelial growth factor (VEGF) and matrix metalloproteinases (MMP)-2 have also been used as targets for neovascularization inhibition in diabetic retinopathy. Several studies have in fact shown that VEGF stimulates the development of abnormal blood vessels in proliferative diabetic retinopathy, while MMPs are involved in chorodial neovascularization [44]. In addition, screening is also based on a cell model, human-derived retinal pigment epithelial ARPE-19 cells that play important roles in the pathogenesis of diabetic retinopathy [45].

## 3. Marine Microorganisms with Anti-Diabetes Properties

Cannell and co-workers, already in 1987, screened 500 freshwater and marine cyanobacteria to detect possible inhibitors of α-glucosidase and α-amylase using colorimetric assays. They found 38 interesting cyanobacteria species containing possible glycosidase inhibitors [46]. More recently, Pandey et al. [30] looked for compounds with inhibitory effects on β-glucosidase in bacteria. This enzyme plays a key role in the degradation of polysaccharides and the processing of glycoproteins and glycolipids, representing a good target for the treatment of diabetes and obesity. Pandey and co-workers [30] found that bacteria associated with the marine sponge, *Aka coralliphaga*, produced a large number of glucosidase inhibitors.

Imada [26] also reported several enzyme inhibitors and other bioactive compounds from marine actinomycetes (e.g., *Streptomyces* sp.). *Streptomyces corchorusii* subsp. *rhodomarinus* showed interesting α-amylase inhibition, while another Streptomyces strain (*Streptomyces* sp.) collected at a depth of approximately 100 m from Otsuchi Bay in Iwate Prefecture, was found to produce two novel compounds, Pyrostatins A and B, with specific inhibitory activity against *N*-acetyl-glucosaminidase.

In addition to bacteria, cyanobacteria and actinomycetes, marine fungi have also been screened for possible anti-diabetic bioactivities [47]. Bioassay-guided investigation of the culture broth obtained from the marine-derived fungus *Cosmospora* sp. SF-5060, isolated from an inter-tidal sediment collected at Gejae Island (Korea), brought to the discovery of the compound Aquastatin A with potent inhibitory activity against the enzyme PTP1B.

Microalgae have also been screened for their anti-diabetic activity. Microalgae are photosynthetic eukaryotes that constitute one of the major components of marine and freshwater phytoplankton [48,49]. Recent advances in aquatic biotechnology have identified a series of microalgal species with promising anti-diabetes properties (Table 1). In 2010, Sun and co-workers [43] evaluated the anti-glycation activities of 20 microalgae during different growth phases. The green microalgae *Chlorella* sp. and diatom *Nitzschia laevis* exhibited the highest inhibitory effects against the formation of total AGEs, especially pentosidine and Nε-Carboxymethyllysine. Using HPLC and gas chromatography analyses, Sun and co-workers [43] revealed that carotenoids (e.g., neoxanthin, violaxanthin, antheraxanthin and lutein) contributed to the strong anti-glycative capacities in *Chlorella* sp., whereas the linoleic, arachidonic and eicosapentaenoic (EPA) fatty acids contributed to the same bioactivity in *Nitzschia laevis*. In 2011, Sun and co-workers [50] tested the anti-glycoxidative properties of different extracts (each extract had different concentrations of the carotenoid astaxanthin) of *Chlorella zofingiensis*. They showed that extracts rich in astaxanthin exhibited higher antioxidant abilities as well as stronger anti-glycative capacities, suggesting that this microalga can be a beneficial food supplement and a possible preventive agent for diabetic patients.

Successively, Sun and co-workers [45] evaluated the protective effects of three microalgal strains (the green algae *C. zofingiensis* and *Chlorella protothecoides*, and the diatom *N. laevis*) against both endogenous and exogenous AGEs in the ARPE-19 cell-based model. In addition, they also tested the major nutritional ingredients present in these microalgae: the carotenoids astaxanthin and lutein, and the omega-3 fatty acid EPA. They observed that the three microalgae as well as their nutritional ingredients attenuated the deleterious effects induced by exogenous AGEs, such as ARPE-19 cell proliferation. The intracellular oxidative stress induced by high glucose levels was significantly prevented by the *C. zofingiensis* extract and by the administration of astaxanthin in a dose-dependent manner [45]. This was expected considering that astaxanthin has an antioxidant activity that is 10 times greater than other carotenoids such as zeaxanthin, lutein, canthaxanthin and b-carotene, and 100 times greater than vitamin E (α-tocopherol) [51]. In addition, the three microalgae and nutritional ingredients reduced mRNA expression levels of VEGF and MMP-2, which are critical steps involved in the pathogenesis of diabetic retinopathy. The authors proposed these microalgae containing high levels of the carotenoids astaxanthin and lutein and the omega 3 fatty acid EPA as beneficial food ingredients and possible preventive agents for patients with diabetic retinopathy and also other ocular diseases, such as cataract and macular degeneration [45].

In 2012, Sun and Chen [31] deeply investigated the anti-diabetes properties of the green algae *Chlorella pyrenoidosa* by evaluating the antioxidant capacity and the effects on two of the key enzymes relevant for type-2 diabetes, i.e., α-amylase and α-glucosidase. The authors showed that *C. pyrenoidosa* had interesting antioxidant activities, inhibiting both α-amylase and α-glucosidase enzymes. The search for antioxidant compounds is of primary interest, since oxidative stress is a major cause of inflammatory events implicated in a large number of diseases (e.g., diabetes, cancer, neurodegenerative and cardio-vascular diseases). Altogether, these studies highlighted the useful anti-diabetes properties of *Chlorella* spp. and this was also taken into account by the biotechnology company Solazyme in the United States which has a patent for the use of *C. protothecoides* to treat people with impaired glucose tolerance and diabetes (US 8747834 B2).

In addition to *Chlorella* spp., Nuño et al. [40] studied the effects of the microalgal haptophyte *Isochrysis galbana* and ochrophyte *Nannochloropsis oculata* on glucose, body weight, lipids, lipoproteins, nitrogen compounds and intestine histopathology in a diabetic rat model. Both microalgae increased the production of low-density lipoproteins and decreased high-density lipoproteins in healthy and diabetic rats. In addition, *I. galbana* promoted body weight loss, decreased glucose, triacylglycerol and cholesterol values and showed only minor signs of intestinal inflammation. The activity may be ascribed to the high quantities of docosahexaenoic acid (DHA) and EPA fatty acids [40]. The *N. oculata* diabetic group exhibited no changes in clinical values and had negative effects throughout the gastrointestinal tract. Further research will be needed to evaluate the possible use of *I. galbana* as an anti-diabetes functional food.

Considering that stress conditions may enhance the production of bioactive compounds [52], recently Ingebrigtsen et al. [53] and Lauritano et al. [52] tested the anti-diabetes properties of several microalgae cultured in stressful conditions, using the PTP1B assay (i.e., evaluation of the Protein Tyrosine Phosphatase 1B inhibition). Ingebrigtsen et al. [53] tested the less polar fraction of 5 North-Atlantic diatoms (i.e., *Attheya longicornis*, *Chaetoceros socialis*, *Chaetoceros furcellatus*, *Skeletonema marinoi* and *Porosira glacialis*) grown in four different light/temperature conditions: high/low temperatures (ranging from 3.3 to 9 °C) and high/low light irradiance (ranging from 30 to 160 μmol photons m^−2^·s^−1^). All *A. longicornis* and *C. furcellatus* extracts were active against PTP1B. *C. socialis* was active only when cultivated at high temperature–low light, while *P. glacialis* in high temperature–high light. *S. marinoi* was not active under any of the conditions tested. These results confirmed that culturing conditions are very important in triggering the production of the bioactives of interest. On the other hand, Lauritano et al. [52] screened crude extracts of 32 microalgal species (21 diatoms, seven dinoflagellates and four flagellates) grown in three different culturing conditions, i.e., replete medium, and nitrogen- and phosphate-starved media (90 μM NO_3_^−^ for nitrogen-starved and 0.5 μM PO_4_^2−^ for phosphate-starved media). Results did not show active hits for the microalgae cultured in these conditions, including 21 diatom species, thereby indicating that temperature/light stress may be more important than nutrient stress in triggering the production of bioactive compounds that inhibit the PTP1B enzyme associated with type-2 diabetes.

## 4. Marine Macroorganisms with Anti-Diabetes Properties

In the last 15 years, several marine macroorganisms have also been screened for possible anti-diabetes properties, e.g., macroalgae, seagrasses, sponges, corals, sea anemones, fishes, salmon skin, a shark fusion protein as well as fish and shellfish wastes (Table 2).

Macroalgae have been consumed as a readily available food especially among coastal communities for centuries, when their nutritional properties and composition were still unknown [36]. Currently, macroalgae are adopted as part of a healthy lifestyle in different countries and are consumed entirely or are used as extracts or food additives [36].

Several red, brown and green macroalgae have shown anti-diabetes properties (e.g., *Rhodomela confervoides*, *Ecklonia cava*, *Palmaria*, *Alaria* and *Ascophyllum*). A bromophenol, 3,4-dibromo-5-(2-bromo-3,4-dihydroxy-6-(ethoxymethyl)benzyl)benzene-1,2-diol isolated from the red alga *Rhodomela confervoides*, and also its synthetic analog 3,4-Dibromo-5-(2-bromo-3,4-dihydroxy-6-(isopropoxymethyl)benzyl)benzene-1,2-diol (HPN), have potent PTP1B inhibitory action in vitro [37,54,55]. HPN also significantly decreased plasma glucose, serum triglycerides and total cholesterol in a mouse model [37]. Two other bromophenols, 2,4,6-tribromophenol and 2,4-dibromophenol, purified from the red alga *Grateloupia elliptica* showed inhibition against *Saccharomyces cerevisiae* α-glucosidase and against *Bacillus stearothermophilus* α-glucosidase [27]. In addition, both compounds inhibited rat-intestinal sucrase and maltase [27]. Besides inhibition against PTP1B and α-glucosidase, some bromophenols also inhibit aldose reductase [33], the first enzyme of the polyol pathway responsible for fructose formation from glucose. For example, bromophenols from the red alga *Symphyocladia latiuscula* have aldose reductase inhibitory activity and could be used in the treatment of complications of diabetes, such as eye and nerve damage in type-2 diabetes patients [54,56]. Phenolic extracts of the red alga *Palmaria* sp. showed inhibitory effects on α-amylase activity [29], while, in another study, protein hydrolysates from *Palmaria palmata* showed potential anti-diabetes properties, i.e., dipeptidyl peptidase IV inhibitory activity [25].

Regarding brown algae, methanolic extracts of the brown algae *Pelvetica siliquosa, Ecklonia cava* and *E. stolonifera* reduced plasma glucose levels in diabetic rats [39,57,58]. Phenolic extracts of the brown algae *Alaria* and *Ascophyllum* exhibited inhibitory effects on α-amylase activity, with *Ascophyllum* also inhibiting α-glucosidase [29]. The phlorotannin Phlorofucofuroeckol-A isolated from *Ecklonia stolonifera* showed significant inhibitory effects against AGEs [59,60]. The phlorotannins Dieckol and Eckol, isolated from *Eisenia bicyclis,* successfully inhibited α-amylase [61], while Diphlorethohydroxycarmalol, a phlorotannin isolated from the brown alga *Ishige okamurae*, showed inhibitory effects against both α-glucosidase and α-amylase [62]. Finally, polyphenol-rich extracts from *Ecklonia cava* [39], *Ulva rigida* and the seagrass *Posidonia oceanica* reduced plasma glucose levels in diabetic rats [63,64].

In 2013, Popov and Krivoshapko [11] studied a total mixture of polar lipids from sea macrophytes *Sargassum pallidum*, *Ulva fenestrata*, *Zostera marina* and a polyphenolic complex from the seagrass *Zostera marina* under conditions of impairments of carbohydrate and lipid metabolism in mouse models. Doses and compositions of the mixtures were optimized in mice with hyperlipidemia and diabetes in order to provide innovative biologically active additives and remedies for metabolic disorders.

Other examples of seaweeds that have shown interesting anti-diabetes properties are *Cladophora rupestris*, able to significantly inhibit α-glucosidase and α-amylase in vitro [28], *Derbesia marina* and *Symphycladia latiscula*, able to inhibit PTP1B in vitro [65], and *Laminaria angustata* Kjellman var. *longissima* (in particular its natural sodium alginate), able to reduce blood glucose levels in Winstar rat model [66]. Sharifuddin and co-workers [36] reviewed beneficial roles of seaweeds for diabetes prevention and management. They highlighted the healthy nutritional composition that may benefit diabetic patients: for example, unsaturated fatty acids, dietary fibers as well as bioactive compounds (see review [36]).

Fucoxanthin, a characteristic carotenoid present in brown seaweeds (and also in some microalgae such as diatoms), is considered a treasure from the sea. D’Orazio et al. [8] demonstrated that fucoxanthin and its metabolites prevented the development of diabetes through down-regulation of mRNA levels of inflammatory mediators, such as TNF-α and IL-6, in a model of obese/diabetic mice. In addition, fucoxanthin promoted the recovery of blood glucose uptake to muscle by the up-regulation of glucose transporter 4, which is also related to the anti-diabetic effects. For these reasons, fucoxanthin is regarded as a potential anti-obesity and anti-diabetic functional food with no known side effects [8].

Marine sponges have been considered as an excellent source of marine natural products since the 1950s, with about 4851 compounds described to date, contributing to nearly 30% of all marine natural products discovered so far [67,68,69,70]. Several sponges show anti-diabetes properties, e.g., inhibition of GSK-3β, α-glucosidase, PTP1B, dipeptidyl peptidase IV or protection of the beta pancreatic cells. A sesquiterpene named palinurin, found in the sponge *Ircinia dendroides*, and a phenylmethylene hydantoins, from the sponge *Hemimycale Arabica,* showed GSK-3β inhibitory activity [24,71]. In 2007, a patent was published on GSK-3β inhibitors from the marine sponges *Ircinia dendroides*, *Ircinia variabilis* and *Ircinia oros* collected from the Mediterranean Sea (US 20070088080 A1).

Callyspongynic acid, isolated from sponge *Callyspongia truncata* inhibited α-glucosidase [72] and the α-galactosylceramide (α-GalCer) from the sponge *Agelas mauritianus* [2,73] induced protection of pancreatic β cells, whereas aqueous extracts of the sponge *Xetospongia muta* inhibited dipeptidyl peptidase IV activity [74] in in vitro models. Inhibitory effects on the enzyme PTP1B have been reported for a polybromodiphenyl ether from the Indonesian marine sponge *Lamellodysidea herbacea* [75] and for the terpene Dysidine, from the sponge *Dysidea* sp. [23,76], that has recently entered pre-clinical trials for the treatment of type-2 diabetes [23]. Dysidine was found for the first time in a sponge at Lahdu (Santo) in Vanuatu in June 1996, identified as sponge *Dysidea* sp. (family Dysideidae, order Dictyoceratida) by Giannini et al. [77]. Successively, Li and co-workers [78] isolated Dynosine from the Hainan sponge *Dysidea villosa* in the Chinese South Sea. The mechanism of action of Dysidine from *Dysidea villosa* was first studied by Zhang and co-workers [76] who found a strong PTP1B inhibition activity. Further cell based evaluation of dysidine indicated that (1) it could strongly promote membrane translocation of the glucose transporter 4 (GLUT4) in CHO-K1 (from Cricetulus griseus ovary) and 3T3-L1 (from Mus musculus embryo) cells, thus indicating the involvement of GLUT4 in the promotion of glucose uptake; (2) Dynosine activated the insulin receptor by modifying its phosphorylation (by PTP1B inhibition). In addition, the cytotoxicity test against Hela cell line (from Homo sapiens cervix) showed no toxicity for this compound [78]. Malve et al. [23] reported this compound in preclinical studies and to our knowledge it is not yet in clinical trials.

Regarding corals, methanolic extracts of two soft corals *Sinularia firma* and *Sinularia erecta* have been shown to exert a blood-glucose-lowering effect in diabetic rats and also inhibited postprandial increase in hyperglycemia in normal rats [41]. Many compounds have been isolated from these extracts; however, none of these have shown a promising inhibitory effect on the tested enzymes of the insulin/glucose/glycogen cascade, i.e., PTP1B and glucose-6-phosphatase. Tiwari et al. [42] investigated the anti-hyperglycemic activity of different sponges and corals. The authors found that extracts of the soft corals *Lobophytum pauciflorum* and *Sarcophyton glaucum*, and the sponge *Sigmadocia pumila* showed some effect in lowering blood glucose post sucrose loads in normal rats, but the effect was not significant. There is also an isolated report that aqueous extracts of the sea anemones *Bunodosoma granulifera* and *Bartholomea annulata* inhibited dipeptidyl peptidase IV activity in in vitro models [74] but to our knowledge there is no further information available on the bioactivity of sea anemones to treat diabetes.

Finally, other marine species have been investigated for potential anti-hyperglycemic and anti-diabetes activities, including fish, salmon skin, a fusion shark protein as well as fish and shellfish wastes. Marine collagen peptides from wild fish decreased free fatty acids and regulated metabolic nuclear receptors in type-2 diabetic patients [79], and *n*-3 PUFAs from fish oil restored insulin receptor and its substrate phosphorylation in Winstar rat models [80] and reduced glucose oxidation and increased glycogen storage in healthy humans [81]. Zhu and co-workers [13] tested oligopeptides obtained from skin of the salmon *Oncorhynchus keta* for possible anti-diabetic effects on rats. The authors found a significant increase in the free-radical detoxification enzyme superoxide dismutase (SOD) and increased serum levels of the antioxidant protein glutathione (for SOD and glutathione functions see [82]) in diabetic rats treated with salmon oligopeptides, suggesting a strong antioxidant activity. The authors proposed this antioxidant activity as a possible protection of pancreatic β-cells from apoptosis [13]. Zhang and co-workers [12] investigated the effects of skin gelatin from the chum salmon *O. keta* on defective wound repair in the skin of diabetic rats. They found that when diabetic rats were treated for 14 days with this gelatin, wound closure improved and there was a reduced inflammatory response suggesting that salmon skin gelatin has beneficial properties for treating wound disorders associated with diabetes.

Recently, Liu et al. [4] evaluated if the cholera toxin B subunit and active peptide from shark liver (CTB-APSL) fusion protein plays a role in the treatment of type-2 diabetic mice. The authors showed that the oral administration of CTB-APSL fusion protein can effectively reduce the levels of blood glucose and glycosylated hemoglobin, promoting insulin secretion and improving insulin resistance. CTB-APSL fusion protein also significantly improved lipid metabolism, reduced triglycerides, total cholesterol and density lipoprotein levels. Furthermore, CTB-APSL improved the inflammatory response in type-2 diabetic mice by reducing the levels of inflammatory cytokines TNFα and IL6.

Finally, considering the huge quantities of underutilized marine processing byproducts as wastes, in recent years, efforts have been made to also test these materials for the treatment of diabetes [83,84]. Fish muscle derived peptides, fish skin collagen and gelatin, fish bone and internal organs, fish oil, shellfish and crustacean shells (in particular, chitin, chitosan and their oligomers) have been screened and used for various biomedical and nutraceutical applications. However, to our knowledge, fish oil was the only one to show anti-diabetes properties (i.e., to accelerate glucose uptake and maintain normal glucose metabolism; [83,85,86]) until now.

## 5. Conclusions

**Figure 2:**
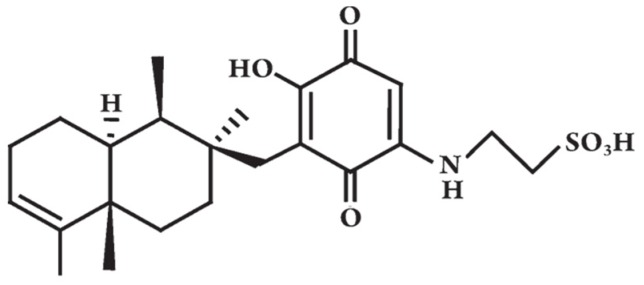
The chemical structure of Dysidine, modified from [76].

Because of the limited number of natural or synthetic anti-diabetic drugs, the search for new possible anti-hyperglycemic and anti-diabetic agents, especially from natural sources, has attracted much interest from the scientific community. As shown in this review, marine micro- and macroorganisms contain biologically active compounds with potential applications as anti-diabetic drugs. Of these, the most interesting compound is the terpene Dysidine extracted from the sponge *Dysidea* sp. (Figure 2), currently in preclinical trials for the treatment of diabetes [23]. The discovery of the first anti-diabetic compound to enter pre-clinical trials certainly will give new impetus to the search for novel ocean medicines for this chronic and important disease affecting such a large sector of the human population.

## Figures and Tables

**Table 1:** Summary of tested microorganisms and possible compounds responsible for the observed anti-diabetes properties (Advanced glycation end-products are reported with AGE, the protein tyrosine phosphatase 1B with PTP1B and not available with N.A.). Main active species names are reported in red.

Species	Possible Compounds	Tested Activity	Reference
500 freshwater and marine cyanobacteria	N.A.	α-glucosidase inhibition	[46]
Bacteria	N.A.	α-amylase and α-glucosidase inhibition	[30]
Actinomycetes Streptomyces corchorusii	N.A.	α-amylase inhibition	[26]
Actinomycetes *Streptomyces* sp.	Pyrostatins A and B	*N*-acetyl-glucosaminidase inhibition	[26]
Fungus *Cosmospora* sp.	Aquastatin A	PTP1B Inhibition	[47]
Three clones of the microalgae *Chlorella pyrenoidosa*, *Chlorella protothecoides*, three clones of *Chlorella vulgaris*, four clones of *Crypthecodinium cohnii*, Nitzschia laevis	Carotenoids, linoleic acid, arachidonic acid, eicosapentaenoic acid	AGE formation inhibition	[43]
Microalgae Chlorella zofingiensis	Astaxanthin	AGE formation inhibition	[50]
Microalgae Chlorella protothecoides, Chlorella zofingiensis, Nitzschia laevis	Astaxanthin, lutein and eicosapentaenoic acid	AGE formation inhibition	[45]
Microalgae *Chlorella pyrenoidosa*	N.A.	Antioxidant potential, α-amylase and α-glucosidase inhibition	[31]
Microalgae Isochrysis galbana, *Nannochloropsis oculata*	Docosahexaenoic and Eicosapentaenoic acids	Clinical values and intestinal inflammation in rats	[40]
Microalgae *Attheya longicornis, Chaetoceros socialis, Chaetoceros furcellatus, Skeletonema marinoi and Porosira glacialis*	N.A.	PTP1B Inhibition	[53]
Several microalgae	N.A.	PTP1B Inhibition	[52]

**Table 2:** Anti-diabetes screening of macroorganisms for bioactive compounds and their mechanisms of action (Advanced glycation endproducts are reported with AGE, the protein tyrosine phosphatase 1B with PTP1B and glycogen synthase kinase 3β with GSK-3β).

Species	Compounds/Extracts	Mechanism of Action	Reference
Red algae *Rhodomela confervoides*	3,4-dibromo-5-(2-bromo-3,4-dihydroxy-6-(ethoxymethyl)benzyl)benzene-1,2-diol	PTP1B inhibition	[37,55]
Red algae *Grateloupia elliptica*	2,4,6-tribromophenol and 2,4-dibromophenol	α-glucosidase, sucrase and maltase inhibition	[27]
Red algae *Symphyocladia latiuscula*	Bromophenols	Aldose reductase inhibition	[56]
Red algae *Palmaria* sp.	Phenolic extracts	α-amylase inhibition	[29]
Red algae *Palmaria palmata*	Protein hydrolysates	Dipeptidyl peptidase IV inhibition	[25]
Brown algae *Ecklonia cava*	Methanolic extracts	Reduce plasma glucose levels in rats	[39]
Brown algae *Pelvetica siliquosa*	Raw extracts	Reduce plasma glucose levels in rats, increase insulin concentration	[57]
Brown algae *Alaria* sp.	Phenolic extracts	α-amylase inhibition	[29]
Brown algae *Ascophyllum* sp.	Phenolic extracts	α-amylase and α-glucosidase inhibition	[29]
Brown algae *Ecklonia stolonifera*	Phlorofucofuroeckol-A	AGEs inhibition	[59]
Brown algae *Ecklonia stolonifera*	Methanolic extracts	Reduce plasma glucose levels in rats	[58]
Brown algae *Ecklonia cava*	Polyphenol-rich extracts	Reduce plasma glucose levels in rats	[39]
Brown algae *Eisenia bicyclis*	Dieckol	α-amylase inhibition	[61]
Brown algae *Eisenia bicyclis*	Eckol	α-amylase inhibition	[61]
Brown algae *Ishige okamurae*	Diphlorethohydroxycarmalol	α-amylase and α-glucosidase inhibition	[62]
Green algae *Ulva rigida*	Raw extracts	Reduce plasma glucose levels in rats	[63]
Seagrass *Posidonia oceanica*	Raw extracts	Reduce plasma glucose levels in rats	[64]
Macrophytes *Sargassum pallidum*, *Ulva fenestrata* and *Zostera marina*	Mixture of lipids, Echinochrome A and polyphenols	Protective effects in mice models	[11]
Seaweed *Cladophora rupestris*	Raw extracts	α-amylase and α-glucosidase inhibition	[28]
Seaweeds *Derbesia marina* and *Symphycladia latiscula*	Raw extracts	PTP1B inhibition	[65]
Seaweed *Laminaria angustata*	Raw extracts	Reduce plasma glucose levels in rats	[66]
Brown algae	Fucoxanthin	Inflammation reduction	[8]
Sponge *Ircinia dendroides*	Palinurin	GSK-3β inhibition	[24]
Sponge *Hemimycale arabica*	Phenylmethylene hydantoins	GSK-3β inhibition, increase liver glycogen in rat	[71]
Sponge *Callyspongia truncata*	Callyspongynic acid	α-glucosidase inhibition	[72]
Sponge *Lamellodysidea herbacea*	Polybromodiphenyl ether	PTP1B inhibition	[75]
Sponge *Xetospongia muta*	Aqueous extracts	Dipeptidyl peptidase IV inhibition	[74]
Sponge *Agelas mauritianus*	α-GalCer	Protection beta pancreatic cells	[2]
Sponge *Dysidea villosa*	Dysidine	PTP1B inhibition	[76]
Corals *Sinularia firma* and *Sinularia erecta*	Methanolic extracts	Reduce plasma glucose levels in rats	[41]
Corals *Lobophytum pauciflorum* and *Sarcophyton glaucum*, and sponge *Sigmadocia pumila*	Raw extracts	Reduce plasma glucose levels in rats	[42]
Wild fishes	Marine collagen peptides	Decrease free fatty acids and regulate metabolic nuclear receptors in type-2 diabetes patients	[79]
Fish oil	*n*-3 PUFAs	Restoration insulin receptor and its substrate phosphorylation in rat	[80]
Sea anemones *Bunodosoma granulifera* and *Bartholomea annulata*	Aqueous extracts	Dipeptidyl peptidase IV inhibition	[74]
Salmon *Oncorhynchus keta* skin	Oligopeptides	Antioxidant activity	[13]
Salmon *Oncorhynchus keta*	Gelatin skin	Wound repair in rat skin	[12]
Shark	Cholera toxin B subunit and peptide shark liver fusion protein	Protective effects in rat model, inflammation reduction, promote insulin secretion, reduce plasma glucose levels	[4]
Fish and shellfish wastes	oil	Lower blood pressure and triacylglycerol concentrations, maintain normal glucose metabolism	[83,84]
